# Stuck in Limbo: how sensemaking discrepancy over strategy-related performance leads to disjointed collaboration in an international joint venture

**DOI:** 10.1007/s10490-023-09877-6

**Published:** 2023-03-28

**Authors:** Xiaoli Zhao, David R. Stiles

**Affiliations:** 1 Dame Mira Szászy Research Centre, The University of Auckland Business School University of Auckland, Private Bag 92019, Auckland, 1142 New Zealand; 2 Department of Management, Marketing & Tourism, University of Canterbury Business School, University of Canterbury, Private Bag 4800, Christchurch, 8020 New Zealand

**Keywords:** International Joint Venture, Sensemaking discrepancy, Collaborative action, Strategy practice, Interpretive approach, Dairy industry

## Abstract

A major issue in international business is why many International Joint Ventures (IJVs) fail to live up to partners’ expectations. Research into why IJVs underperform centres on differences between partners’ equity, resources, technical knowledge and cultural values, but seldom internal sensemaking conflicts. We address this research gap: specifically, the sense managers make of their own and their partner managers’ perceived performance in relation to strategy practices, and the effects of sensemaking upon collaboration. Some IJV studies examine outright organizational failure, but we focus on a common situation where partner firms’ expectations about each other’s performance are not met. Our case is a major Sino-New Zealand dairy IJV in a Limbo-like state of severe sensemaking discrepancy. Here, managers struggled to perform strategy effectively in a context of mutual misunderstanding and profound miscommunication, rooted in sensemaking differences. Using a strategy practice lens, we explore how this sensemaking discrepancy arose over organizational identity, learning and experience, strategizing, communication and trust. This eroded meaningful cooperation over strategy, leading to *disjointed collaboration*: a new concept capturing a state of compromised engagement, where the IJV continued operationally, but collaboration became increasingly difficult. We provide a theoretical framework to help understand sensemaking discrepancy in IJVs, based on a reconceptualization of sensemaking discrepancy in terms of *own and others’ expected and perceived performance*. We also offer essential practice-based insights into cognitive barriers to strategy collaboration.

One of the most pressing problems in international business is why many cross-border collaborations fail to live up to partners’ expectations. Some dysfunctionality may be tolerated, but what happens when partners’ expectations of each other’s strategy performance are largely unmet? This study examines how international joint venture (IJV) managers make sense of their own and their partner managers’ strategy-related performance during collaboration. We show how severe discrepancy arises in sensemaking about the IJV’s identity, organizational learning, strategizing, communication and trust, leading to a Limbo-like state of underperformance.

Although reported survival rates for International Joint Ventures (IJVs) vary between 40 and 70% (Yang, 2011), many IJVs experience substantive conflict over strategic and operational decisions without necessarily failing (Ding, 1997; Liu et al., 2014). Challenges arise from partners’ disagreements over equity holdings (Li et al., 2009), resource asymmetries (Child & Yan, 1999), technology and knowledge transfer (Pak et al., 2015), and broad differences in cultural values (Barkema & Vermeulen, 1997). We know partners’ expectations of one others’ performance are important in rapidly changing environments (Maitlis & Christianson, 2014), but we do not know how mutual expectations over strategizing evolve. There is a clear research gap in understanding the sensemaking processes contributing towards IJV difficulties, whether leading to outright failure or not.

Sensemaking about international strategy practices is an underdeveloped research stream (Jalonen et al., 2018; Rouleau & Balogun, 2011). Conflicts may arise from discrepancy in the sense IJV partners make of each other’s strategy activities, but scholars believe these are often resolved as actors develop congruent sensemaking schemes (Das & Kumar, 2010a; Gertsen & Søderberg, 2011). At other times, it is thought that sensemaking differences become more intractable (Das & Kumar, 2010b), but theory is embryonic and little empirical support exists.

We therefore explain how sensemaking discrepancy between IJV managers may arise and intensify. This is based on individual managers comparing their own strategy-related performance with that of their partner managers. Our case shows increasing sensemaking discrepancy between partners, resulting in severe collaboration issues. In Milton’s (1667) portrayal, ‘Limbo’ is a figurative sense of a place for people and things forgotten. This rich metaphor appropriately describes a venture seemingly lost in a state of mutually disappointed expectations.

This study is important because it shifts focus away from economic or broad cultural explanations of IJV issues towards a sensemaking one. We respond to calls for ‘thick’, phenomenological work (Nippa & Reuer, 2019; Welch et al., 2011) on partners’ collaborative practices. The study examines sensemaking accounts relating to strategy in a dairy IJV, ‘MILK’, involving Chinese (‘CHD’) and New Zealand (‘NZD’) dairy company partners: all pseudonyms to preserve confidentiality.^1^ New Zealand dairy companies are significant players in the global dairy industry (Basset-Mens et al., 2009), and the case is a prominent China-based IJV.

We first identify a research gap concerning IJV sensemaking about strategy practices, then explain our method for comparing partners’ strategy-oriented perspectives. Our case reveals dimensions of *sensemaking about identity, learning, strategizing, communication and trust*. It also yields insights into how tensions arise from a developing sensemaking discrepancy in terms of managers’ *own prospective performance and* their partner managers’ *expected and perceived performance*, leading to a new concept of *disjointed collaboration*: a Limbo-like state of compromised engagement in which the IJV continues operationally, but actors find mutual understanding and strategy cooperation increasingly problematic.

## International joint ventures and sensemaking about strategy practices

### International joint ventures

An IJV is an equity form of alliance owned by two or more corporate ‘parents’ from different countries aiming to pool capital, share risks and seek synergies from a combination of resources and capabilities; with no partner controlling all activities (Huang & Chiu, 2014). IJVs are the dominant form of alliance in China, because overseas partners face investment restrictions; and many ventures are seen as longer-term strategic collaborations (Liu et al., 2014). Partners aim to create sustained economic value by accessing new local and international markets and networks, raw materials, technologies and cost-effective manufacturing or services (Johnson et al., 2006), and benefit from mutual learning (Park, 2011).

However, collaboration may be adversely affected by conflict over equity, resource asymmetries and knowledge transfer. Struggles over ownership are common (Choi & Beamish, 2004; Li et al., 2009): some partners seek a majority shareholding to increase strategic control (Ding, 1997), while others favour more balanced holdings to enhance mutual trust (Beamish & Lupton, 2009). Asymmetries develop because many resources are firm-specific and not transferable or imitable (Child & Yan, 1999). Conflicts arise from attempts to control transaction costs (Beamish & Banks, 1987) and interdependencies (Lioukas et al., 2016), with partners seeking returns on resource contributions, market knowledge, and research and development (Huang & Chiu, 2014). Knowledge transfer (Pak et al., 2015) creates issues as partners try to protect their own technology, skills and tacit knowledge in the face of information ambiguity (Ho et al., 2019), opportunistic behaviour (Liu et al., 2014) and disagreements over sharing innovation risks (Park, 2011) and local networks (Kim & Kim, 2018).

Others attribute difficulties to broad cultural differences, as measured by national values surveys (Hofstede, 1980; Trompenaars & Hampden-Turner, 1998; Javidan & House, 2001). Dissimilar national origins hinder IJV survival (Barkema & Vermeulen, 1997), with firms encouraged to develop transnational organizational cultures (Pothukuchi et al., 2002) to ensure better strategic ‘fit’ and economic performance (Meirovich, 2010), or more localized strategy (Reuer et al., 2014). Cultural explanations extend the argument beyond an economic- or knowledge-based focus (Blodgett et al., 2008), but fail to explain the reasoning, characteristics and outcomes of managerial decision-making (Aharoni et al., 2011). They also ignore within-country cultures (Taras et al., 2016) and increasingly complex multicultural and hybrid cultures (Johnson et al., 2006). Many studies disregard local and foreign partners’ behaviour (Delios, 2017). In general, deeper contextualisation is needed (Szkudlarek et al., 2020).

Intercultural perspectives provide useful insights into cultural-fit between partners (Froese et al., 2012), cross-cultural competences (Griffith, 2003), language misunderstandings (Wang et al., 2020) and misaligned communication (Zhao & Mills, 2019). These reveal richer views of culture than values surveys alone (Osland & Bird, 2000), but studies lack consistent methods (Moore, 2011) and neglect internationalisation shifts (Birkinshaw et al., 2011) and more complex intercultural communication (Szkudlarek et al., 2020). More contextualized studies on alliances and multinational enterprises are emerging concerning biculturalism (Moore, 2011); subsidiary-parent relationships (Balogun et al., 2011); subsidiary narratives (Gertsen & Søderberg, 2011); and legitimacy-building for controversial decisions (Balogun et al., 2019). Yet, none of these examines sensemaking about strategy in IJVs.

### Sensemaking about strategy practices

Applied within a broader strategy-as-practice (SP or SAP) approach, sensemaking offers a rich, contextualized alternative to resource-based, structural or cultural views. SP sees strategy activity as something people *do* or *say* (i.e. their strategy-related activities), rather than as something an organization *has* (i.e. a property of the organization), allowing a finer-grained view of strategy-related activities and interactions (Whittington, 2006; Sandberg & Tsoukas, 2011). SP hones in on an individual’s (micro-level) perceptions and actions (practices) embedded within a web of organizational and social practices (Whittington, 2006), rather than general decision-making patterns (Burgelman et al., 2018). Such approaches reveal the ‘substructure beneath the busy surface of events’ (Vaara & Whittington, 2012: 288). Practitioners are all those ‘who do strategy work’, not just top managers; a practice is ‘a repeated action’ in strategizing; and praxis refers to the flows around ‘doing strategy work’ (Vaara & Whittington, 2012: 286). Actors engage with material artifacts such as tools (Jarratt & Stiles, 2010), instruments or products (Kaplan, 2011) during decision processes concerning an organization’s mission, direction, structure, product-markets or competitive positioning.

A growing sensemaking stream within SP (Burgelman et al., 2018; Jalonen et al., 2018) regards sensemaking as an ongoing process by which people make experience meaningful (Weick, 1995). It embraces the ‘processes of interpretation and meaning production individuals and groups use to reflect on phenomena’ (Brown et al., 2008: 1038). S*trategic sensemaking* is ‘A social process of meaning construction and reconstruction through which managers create sense for themselves and others about their changing organizational context and surroundings’ (Rouleau & Balogun, 2011: 955). It is about people’s attempts to understand past, present, and future situations, through actively constructing a subjective understanding of ‘reality’ to take further actions (Stensaker & Falkenberg, 2007; Weick, 1995) and develop strategy collectively (Seidl & Werle, 2018). Sensemaking is a response to unexpected, novel, or confusing circumstances or events (Maitlis & Christianson, 2014); and includes attempts to influence others’ meaning construction processes through language, symbols, images and material tools (Arnaud et al., 2016) towards a preferred redefinition of organizational reality (Brown et al., 2015). When concerning an anticipated future common in strategizing, it is termed ‘prospective’ sensemaking (Brown et al., 2015; Sandberg & Tsoukas, 2015). While involving routine strategy activities (Arnaud et al., 2016), it is also about major crises such as organizational restructuring (Sandberg & Tsoukas, 2015).

One study links SP and sensemaking to domestic alliances, to show how diversity in participants helps sensemaking handle complex environments (Seidl & Werle, 2018). Other work on domestic collaboration examines how coopetition frames develop (Lundgren-Henriksson & Kock, 2016); how sensemaking processes lead to particular norms of justice in a post-merger integration (Monin et al., 2013); and sensemaking about the Ethiopian business environment (Woldesenbet & Storey, 2010). Only one focuses on IJVs, finding older Russian IJV managers engaged in more traditional centralised planning practices (Kobernyuk et al., 2014). Sensemaking research on IJVs in China is lacking, despite IJVs being the dominant foreign entry mode (Delios, 2017).

Little is known about how IJV managers maintain and develop IJV collaboration – and what happens when this goes wrong in terms of cognitive rather than financial (Tsang, 2016) impacts. We see strategy performance as a set of social practices (Vaara & Whittington, 2012) in which knowledge is developed by individuals in the process of achieving desired outcomes (Dey & Steyaert, 2007). SP research shows strategizing does not just involve senior managers, but also middle and lower managers (Balogun & Johnson, 2004). All perform the strategic conversation by drawing on symbolic and verbal representations and sociocultural systems to determine what to say to stakeholders, crafting and diffusing messages, staging conversations and relating to others (Rouleau & Balogun, 2011). In an IJV, sensemaking activities may be important in how each partner’s managers judge their counterparts’ strategizing performance (Das & Kumar, 2010b); but we do not know how. Therefore, our first research question is:

How do managers from different partners in an international joint venture make sense of their own and each other’s performance in relation to strategy practices during collaboration?

The limited research on sensemaking in IJVs also suggests partners embedded in different national cultures rely on interpretive schemes to make sense of conflicts emerging between them (Das & Kumar, 2010a). Such conflicts may arise at strategic or operational levels and be cognitive or behavioural. At times, this sensemaking discrepancy is believed to influence collaboration more severely (Das & Kumar, 2010b). Collaboration issues are sometimes attributed to a lack of understanding between actors as a consequence of inadequate communication; but we look beyond everyday technical ‘translation’ issues to those rooted in social differences and contextual impediments to understanding (Hong et al., 2016). Different alliance motives and practices may create confusion and loss of goodwill, resulting in fewer synergies than expected and asynchronous collaborative behaviour (Hitt et al., 2000).

IJVs may be particularly prone to such conflict, given the close proximity of partners from different national cultures (Wang et al., 2020). Opportunism, distrust, strategic incompatibility and poor integration may arise from partners interpreting and responding to each other’s behaviour in contradictory ways (Das & Kumar, 2010b). Language differences may also affect perceptions of trustworthiness (Tenzer et al., 2014), although such difficulties are often temporary (Gertsen & Søderberg, 2011; Seidl & Werle, 2018). Usually, predictability remains the systemic operating norm and conflicts can be managed instrumentally through collecting more information, analysis/planning and changing behaviour – a model described by Das and Kumar (2010a, 2010b) as *sensemaking of chaos*. Although cultural and social differences remain, partners develop congruent sensemaking schemes, and the IJV is able to function effectively.

However, ideas may not simply be ‘lost in translation’ but more fundamental *sensemaking discrepancy*, or contradictory meanings, arise in which partners remain unable to make appropriate sense of each other’s orientations and practices. This second model of *sensemaking in chaos* (Das & Kumar, 2010a, b) suggests sensemaking differences can become more intractable where unpredictability is seen as normal and inevitable. Partners may try to demonstrate their commitment symbolically through experiment and incremental decision-making, but are unable to reach a consensus about their respective obligations. Should behavioural and structural contradictions not be effectively managed, the IJV may become unstable and even dissolve. This second model provides an initial scenario for more intractable sensemaking effects, but there is scant research on how sensemaking in chaos occurs. Therefore, our second research question is:

What are the effects of severe discrepancy between partners’ sensemaking processes on collaboration in the international joint venture?

Next, we explain our method for applying a sensemaking perspective to a case study of how sensemaking impacts upon IJV strategizing.

## Research design

Our interpretive approach aimed to understand sensemaking in a case study context (Brown & Humphreys, 2003). Although informed by narrative perspectives, we were concerned less with the structure of storytelling and individuals’ accounts of turning points (Gertsen & Søderberg, 2011) and more with how individuals made sense of their own and others’ strategy practices through *sensemaking accounts*: “Those processes of interpretation and meaning production whereby individuals and groups reflect on and interpret phenomena and produce intersubjective accounts” (Brown, 2000: 3). We focused on sensemaking accounts in interviews about strategy role performance i.e. how IJV actors performed (i.e. acted out) their roles concerning strategy and the judgements they made about their own and others’ performance. Interview data were regarded as politically and identity-loaded; with participants attempting to construe their experiences as legitimate knowledge (Brown et al., 2008; Mills et al., 2010).

We concentrated on sensemaking discrepancy arising once collaboration developed, rather than events prior to the IJV’s establishment - although we refer to these where relevant. Research involved an iterative process whereby data collection and analysis informed each other, giving us confidence our process fully captured participants’ sensemaking interpretations (Lundgren-Henriksson & Kock, 2016).

### Background

The Sino-New Zealand dairy IJV, MILK, was selected ‘purposefully’ rather than at random (Eisenhardt & Graebner, 2007), because access required extensive negotiation. The case was chosen because parent firms from two different countries were juxtaposed, potentially providing a rich context for sensemaking differences (Das & Kumar, 2010a). MILK fitted the minimum criterion of two years’ establishment to provide meaningful data – with the New Zealand (NZ) parent established for 12 years and its Chinese counterpart 64 years prior to the IJV.

China’s rapid growth in household incomes presaged a surge in demand for dairy products, attracting considerable investment from NZ’s dairy industry (Fuller et al., 2006). Lacking scale and modern, pasture-based production methods, and driven by crises in food safety and quality,^2^ Chinese firms looked to foreign partners (Xiu & Klein, 2010). NZ dairy firms are important to the global dairy industry: approximately 70 firms have notable international market shares (Basset-Mens et al., 2009).

The privately-owned NZ parent company (NZD) is a pioneering venture in China, with substantial learning likely for future entrants. NZD is small in relation to the Chinese parent (CHD), with 156 and 1500 employees respectively; but NZD’s shareholding gives it substantive influence in the IJV, as does its milk resources, processing technology, market image and distribution expertise. CHD’s ownership layers provide extra decision-making complexity: alongside many Chinese enterprises, CHD is majority (54%) state-owned as part of a large, centralised food company Group, with the Group Chairman a senior government official.

MILK began life as a NZD subsidiary, before the need for innovation and growth capital meant selling shares to CHD in a converted IJV in 2008, three years after start-up. The case concerns partners’ strategy practices between 2008 and 2016. Neither company had prior experience of operating an IJV. The alliance aimed to access a large potential Chinese market in liquid milk and milk solids such as infant formula. A venture between NZD and CHD appeared a good strategic move, given NZD’s growth plans and CHD’s requirement for technology and safe milk products.

### Data collection

Our larger project collected data from multiple sources - interviews, observations and document analysis (Balogun et al., 2003) – but this study examines sensemaking accounts embedded in interview answers. Observations and archival data were used only to develop interview questions.

A SP lens involves ‘drilling down’ to include senior, middle and junior managers involved in strategy (Vaara & Whittington, 2012: 286), using a ‘thick description’ of strategy practices (Welch et al., 2011). We therefore collected data from managers across all levels, but were less concerned with variations between these levels and more with sensemaking similarities and differences between partners. To avoid pre-structuring ideas about what constitutes ‘strategy’, we asked participants to identify IJV strategy-related activities. The first author, fluent in English and Mandarin, undertook and transcribed 52 in-depth, semi-structured interviews in both languages, with independent back translation ensuring data reliability.

All interviews were designed to encourage free discussion around participants’ sensemaking of events and activities (Taylor, 2011). Questions centred on how participants retrospectively made sense of the way the IJV was structured and operated and the nature and performance of management roles. Data collection was in three interconnected phases, with questions tailored to each phase and participant.

The first phase focused on senior partners’ *individual accounts* of deliberate collaborative action during the IJV’s start-up and early strategizing, including rationales for forming the venture and how collaboration developed. Interviews were held with the seven top executives responsible for establishing and directing the IJV: three at the NZD parent’s headquarters in New Zealand and four at CHD’s main site in China.

Phase two considered senior, middle and junior managers’ strategy-related roles and actions once the IJV was established. Here, 30 semi-structured interviews involved all managers from both sides (20 from NZD and 10 from CHD) who identified with strategy-related activities. Managers described their own and others’ practices during collaboration, producing individual accounts. Specific questions concerned the nature of the IJV relationship, including how this was maintained and developed, with participants asked to recount specific episodes of collaboration. Interviewees self-reported strategizing practices, using their personal experiences to explain how major IJV decisions were made.

This second round of individual accounts helped form *composite accounts* of strategizing practices within each of the two sets of partner managers. A composite account is ‘A novel method to re-present narrative data and qualitative research findings through comparing and contrasting first person accounts’ (Wertz et al., 2011: 1) to construct shared narratives for each IJV partner. Since individual accounts revealed conflicting accounts and highly sensitive data about the IJV relationship, composite accounts helped protect individuals’ identities, while capturing core narratives. Because most individuals’ narratives are (micro) fragments of (meso) organizational stories (Sonenshein, 2009), shared organizational narratives based on partner groupings help connect micro and meso levels (Seidl & Whittington, 2014), while extracts from individual accounts allow richer understandings of the texture and structure of the phenomena and multiple voices (Todres, 2008).

The third phase required participants to engage more deeply in sensemaking by reflecting upon the narratives produced in the first two phases and describing the sense they made of the practices articulated. Data helped develop *intersubjective accounts* (Brown, 2000: 45 − 6). These are ways of dealing with positionalities (including bias, misunderstandings, stereotyping, data loss and power relations), impressions and representations of multiple parties in a research process. This is particularly important where one is trying to capture all voices including those potentially marginalized in a hierarchical context and/or where there are two or more languages or cultures, with interpreters/ translators adding an extra filter to knowledge – known as ‘triple subjectivity’ (Phillips, 2016: 27). Both conditions applied to our case, where junior and middle managers’ accounts were *as* important to sensemaking as senior executives’ (and, as data shows, hierarchy being particularly pronounced in CHD); and two juxtaposed languages/ cultures being interpreted by managers and researchers. Thus, intersubjective accounts accommodated multiple voices and helped validate data further in achieving a combinatory balance between emic (‘insider’ participant) and etic (‘outsider’ researcher) accounts (Phillips, 2016: 30).

This final phase comprised 15 sensemaking interviews: 8 with NZD (4 senior managers and 4 middle managers) and 7 with CHD counterparts. New questions included why partner firms appeared to have a different strategic focus, the main collaborative problems emerging, and reasons for apparent sensemaking difficulties, all centred on specific episodes. Participants were asked to account for their own and their partners’ behaviour, and describe how/ whether disagreements were resolved, the nature of inter-firm/ board meetings, and how and why shareholdings changed.

### Analysis

Analysis focused on sequential collections of events with causal explanations or ‘plots’, examining how actors defined each event and why it occurred (Brown et al., 2008). As in other sensemaking studies (Jalonen et al., 2018), direct transcriptions were used, with data coded iteratively using NVivo software. In the findings and tables, interview extracts are presented with double quotation marks. All other instances are reported or composite/intersubjective themes. We compared coded data from individual accounts of formative, executive collaboration (phase one) and later, more widespread collaboration (phase two) to develop composite accounts of IJV events, activities, strategy practices, and policy changes (Dyer & Wilkins, 1991). The first author grouped candidate themes within these into ‘S’ (similar) and ‘C’ (contrasting) categories of collaboration experiences, comparing these with interview transcripts to ensure ‘meaning coherence’ (Sandberg & Tsoukas, 2015). These were then cross-checked to ensure interpretations captured participants’ main perspectives. The rest of the research team each independently verified initial codes. Coding disagreements were resolved by the researchers re-considering evidence in an iterative process until a consensus was reached. Although time-consuming, this process meant a third party was not needed as a final arbiter.

In phase three, we used themes from composite narratives during our final 15 sensemaking interviews to help managers produce intersubjective accounts. Participants were confronted with their own and partners’ composite narratives and asked to provide reflective, retrospective accounts of sensemaking rationales. Rather than confirming/ refuting strategy practices already identified, participants were encouraged to consider whether any actors were marginalized and whether actors’ positionality may have influenced data, including the researchers’ interpretations (Phillips, 2016). This helped minimize research bias and ensure data validity (van der Giesen et al., 2021).

Table 1 gives examples of this coding process, although the next section provides more comprehensive analysis. The table shows senior managers expressed fundamentally different views of their partner firm’s strategic purpose. Illustrative quotes highlight these differences in the phase one summary. NZD senior managers saw CHD as an important but unequal trading partner, and the IJV as a means to reach Chinese customers. In contrast, CHD senior managers viewed both NZD and the IJV as their subsidiaries, useful mainly in supplying CHD with quality milk and infant formula for the Chinese market. Each party believed the other to be naïve or inexperienced, with NZD managers believing they controlled the company strategically, but CHD executives feeling their partners did not understand the Chinese market.

**Table 1:** Examples of the three stage account analysis

Phase 1	Individual Accounts	
	NZD Senior Managers “[CHD] provides us with valuable strategic insight, particularly in respect of the Asian dairy market, and actively encourages and supports the development of our direct relationships with our wider customer base in China.” (Thomas, Product Development Manager) “[CHD] is one of our important customers. It owns and markets [branded infant formula] products in Shanghai and adjacent provinces which [NZD] manufactures.” (Peter, Sales Manager) “[CHD] provides us money and thinks they own the company. It’s very naïve. We control the company and don’t give them a chance to manage our company.” (Jack, CEO)	CHD Senior Managers “[NZD] is not only a significant investment for [CHD], but also the only supplier of our first high-end infant formula product. [NZD] is important to the development of [CHD’s] whole infant formula business. With the excellent quality product provided by [NZD], together with the developed marketing and distribution business of [CHD], we are confident that our product will position us well in the high-end infant formula market.” (Lu, Strategy Development Manager) “[NZD] is our first subsidiary overseas, so we have paid a lot of attention and given them a lot of support to develop the products.” (Feng, CFO) “We have to do everything to promote the products selling in China as they don’t have any experience.” (Peng, Marketing Manager) “[Jack] is young and ambitious but has little business experience.” (Li, CEO)
	**Summary (selected phase 1 data)** • NZD senior managers consider CHD a trading partner, not an equal IJV partner • NZD senior managers see the main benefit gained from the collaboration as helping NZD develop relationships with China-based customers	**Summary (selected phase 1 data)** • CHD senior managers invested in MILK with the strategic purposes of developing their high-end infant formula business in the Chinese market and accessing NZ milk resources • CHD senior managers regard MILK & NZD as their subsidiaries
Phase 2	Composite Accounts	
	NZD All Managers ***Aspects of IJV strategy practices*** • NZD senior managers attempt to control MILK’s strategies, despite knowing CHD’s majority shareholding gives them the right to do so • CHD’s senior managers do not speak English, so NZD’s senior managers do not try to communicate with them • NZD managers do not want to do as CHD managers require, such as providing them product and milk resource information • NZD senior managers formulate MILK’s strategy in line with NZD’s strategic plan ***IJV collaboration challenges*** • It is increasingly difficult to align NZD strategy ideas with CHD, because CHD managers do not believe their counterparts know how NZ (Western) business works • NZD middle managers feel it is difficult to handle both their own senior managers and those from CHD when they have to get urgent jobs done	CHD All Managers ***Aspects of IJV strategy practices*** • CHD senior managers believe NZD executives are playing a game – sending multiple English-language documents to CHD directors only 2–3 weeks before a board meeting • CHD’s senior managers ask their own middle managers to deal with NZD senior managers because they consider NZD to be their subsidiary • CHD’s marketing team focuses on its own product development strategy when MILK fails to meet its product supply needs • As long as NZD and MILK increase sales revenue and provide CHD with some milk resources, CHD lets NZD implement its own strategies at MILK ***IJV collaboration challenges*** • It becomes more difficult for CHD managers to develop a trusting relationship with NZD managers, since NZD managers believe they are more competent than CHD’s managers • Middle managers follow senior managers’ orders, but cannot do their tasks on time
Phase 3	Intersubjective Accounts	
	NZD All Managers *Identity* • NZD managers are proud of NZD’s product quality & “being smarter” in a short time • CHD is an important customer & shareholder, but NZD senior managers see it as a functional trading partner to reach the Chinese market rather than a relational partner • Despite being an IJV, MILK is really NZD’s investment vehicle, with NZD senior managers aiming to control and manage it, deliberately excluding CHD managers from major decision-making *Learning & experience* • All long-staying NZD managers believe in self-directed learning and responsibility at all managerial levels • NZD senior managers think their strategic capabilities in technology, production and management knowledge and experience are far more advanced & that CHD senior managers do not understand the Chinese market or know how to develop it • NZD senior managers believe themselves superior in personal capabilities to their CHD counterparts, who have inflated self-perceptions of their own capabilities; so, NZD senior managers reject their “interference” *Strategizing* • NZD senior managers believe equity holdings are not central to control of MILK; instead using CHD managers’ perceived poor English and lack Western business & legal system/contractual knowledge s/written to gain control over MILK strategy • NZD senior managers and board directors make their own strategic decisions, unconstrained by other external accountabilities • NZD senior managers hold regular strategy meetings, consultant-led workshops, believing in more formal, hands-on strategizing practices • NZD senior managers prepare discussion documents for board meetings and lead meetings to formulate MILK strategy in line with NZD’s overall strategic planning, as well as slowly reducing CHD’s ownership and authority • NZD senior managers pursue their own strategy objectives to develop several Asian markets for premium, low-volume, sustainable dairy products *Communication & trust* • NZD board members & senior managers try to “push” or “convince” CHD board members to agree with or accept their ideas • Mid- and junior NZD managers see no effective inter-partner liaison mechanisms, listening, or strong personal relations; so, it is difficult to communicate or build trust with CHD managers at all levels • NZD senior managers complain about lack of English language competency among CHD senior managers; but believe they can second-guess CHD senior managers and directors’ body language • NZD middle managers who speak Mandarin are asked to deal with senior managers of CHD, despite the latter being reluctant to talk directly talk to them • NZD managers enforce formal trading contracts, after a breakdown of trust results from CHD managers attempting to renegotiate pricing terms • NZD senior managers provide CHD only with the information they believe necessary to operate MILK as a deliberate strategy to avoid their partner’s “interference”	CHD All Managers *Identity* • CHD managers are proud of their state-owned-enterprise’s heritage & longevity • CHD senior managers see NZD as CHD’s factory and MILK as its subsidiary for overseas expansion: a “new-born” baby needing their guidance • Because CHD owns 51% of shares in MILK, CHD senior managers believe MILK should supply them with NZ dairy resources at the lowest price *Learning & experience* • All CHD managers believe senior people are responsible for teaching and guiding junior members; in turn, junior people respect their seniors • CHD senior managers think they are responsible for teaching and directing all NZD managers about China • CHD senior managers see themselves as superior in personal management capabilities to NZD counterparts, who are unwilling to listen or accept their help *Strategizing* • CHD senior managers perceive a majority MILK equity holding is key to controlling the IJV; and that relational aspects are more important than written contracts • CHD senior managers and board directors defer to the corporate Group and government officials for strategic decisions • CHD senior managers and board directors pay little attention to internal formal strategy processes or board meetings, which they see as more about implementation, believing instead in pragmatic, informal and action-oriented strategizing practices • CHD senior managers pursue their own strategy objectives to produce low-priced, high-volume dairy products initially for Chinese consumers via CHD as their ‘factory’ • All CHD managers actively promote other infant formula products in the Chinese market instead of co-branded products when NZD delays production, resulting in a lack of differentiation *Communication & trust* • CHD senior managers perceive NZD counterparts do not listen to or trust them • CHD senior managers try to adopt a “carrot-and-stick” approach to show both support of and dissatisfaction with NZD’s performance (e.g., give an offer of a one-year executive order for the IJV start-up as encouragement; thump their fists on the boardroom table to show their displeasure and later withdraw financial support as punishment) • CHD senior managers deliberately implement hierarchical communication to show that they regard their own middle management team as equal to NZD’s senior management team; also expecting NZD senior managers to implement decisions and report their outcomes to CHD middle managers • When CHD senior & middle managers are deliberately ignored by NZD senior managers, CHD middle managers ask their juniors to liaise with NZD managers instead • When NZD becomes aggressive (pushing) and the CEO of NZD is purposely distant, CHD senior managers stay back to observe and “maintain harmony”, waiting for a chance to give the CEO a lesson

Composite accounts in Table 1 provide further insights into collective strategizing from phase two interviews. NZD managers felt they controlled IJV decision-making despite CHD’s majority shareholding, avoiding communicating with and providing product information to their counterparts, and applying their own strategy to MILK. CHD senior managers believed their partners were game-playing in not translating board documents in time for meetings and delegating liaison activities to middle managers. CHD managers pursued their own raw material supply when MILK’s was insufficient. As the next section details, collaboration challenges were articulated by both ‘sides’, with strategic alignment, trust and cooperation increasingly difficult to maintain because of contrasting views of each other’s strategic purposes.

Phase three intersubjective accounts confirm this growing disjunction, with NZD board members rationalising lengthy attempts to cajole their Chinese counterparts into agreements, blindsiding them over lack of language ability and western business understanding, restricting the flow of information and attempting to control the strategic agenda. CHD counterparts articulated a ‘carrot and stick’ approach, by offering access to Chinese markets, while expressing concern over their partners’ commercialism. CHD executives adopted a paternalistic management style, resorted to hierarchical communication, and replaced co-branded products with their own. Intersubjective accounts are summarized as themes in Table 1 rather than individual quotes, since they are summaries of participants’ and researchers’ reflections on composite accounts (Phillips, 2016), although quotes from third phase interviews are identified in the “Findings” section.

Table 2 shows a basic coding tree. First order codes are themes emerging from both individual and intersubjective accounts, since both comprise individual-level data. As stated, composite accounts aggregate individual data from phase one and two as inputs into phase three of the research process, so are not part of the coding process. First order codes are grouped into fourteen second-order concepts comprising contrasting sensemaking about pride, organizational purpose, management capabilities, work-based learning, ways of doing business, management capabilities, superiority, equity holdings, contractual practices, corporate strategy independence, strategy process responsibility, strategic decision-making, collaborative contact, communication, and trust. These codes are then aggregated into the final sensemaking categories: sensemaking about identity, learning and experience, strategizing, and communication and trust. The next section details the main findings from the study.

**Table 2:** Coding tree

First-order indicators	Second-order concepts	Themes
• Managers feel a keen sense of pride with both parent companies, with NZD managers basing this on personal achievement and CHD managers on parent company history • Both NZD and CHD managers believe MILK is their own subsidiary, not their partner’s • CHD managers see NZD as their factory, while NZD managers believe CHD is their trading partner in the Chinese market • CHD senior managers believe NZD is a young company and needs to build a solid foundation, otherwise it will grow too fast • CHD managers believe they focus on people and harmony, so key NZD leaders should build the MILK organizational culture first. However, NZD managers do not consider CHD will support them to grow the company culture towards profitability • CHD managers see NZD managers caring more about their personal lives and freedom than about work ethics of diligence, responsibility, and collaboration	• Strong pride in parent firms • Misaligned mission expectations about parent firm & MILK • Divergent cultural expectations	Sensemaking about identity
• NZD managers learn through their own experience and practice, including from making mistakes; CHD middle and junior managers learn from senior managers, transferring taught experience to practice, but with low tolerance for major mistakes • NZD managers say they contribute to the growth of MILK while the Chinese board directors just attend board meetings twice a year without any real contributions • NZD managers express concern that CHD managers try to order them about, which shows naïve business behaviour • NZD middle managers find it difficult to handle both their own and CHD senior managers, while CHD middle managers follow their own senior managers’ orders but cannot get tasks done on time	• Contrasting approaches to work-based learning • Misaligned management capabilities • Lack of mutual learning due to competing senses of superiority	Sensemaking about learning and experience
• NZD managers perceive it makes no difference to CHD executives whether they hold a majority shareholding or not because they lack understanding of Western ways of operating businesses (e.g. They have little sense of written contracts) • NZD senior managers consider CHD managers made a major strategic mistake when allowing their shareholding to be watered down from 51–39% rather than investing further in the IJV • CHD managers believe there is no need to invest more money and effort in the IJV because NZD managers do not keep their promises (e.g. To increase sales volumes and develop new products specifically for the Chinese market)	• Contrasting perceptions of equity holdings • Functional versus relational views of contractual practices	Sensemaking about strategizing
• NZD senior managers enjoy autonomous corporate strategy responsibility; CHD senior managers are subservient to external Group and government stakeholders and are responsible for making sure the IJV’s strategic orientation is consistent with that of the CHD Group • NZD senior managers are involved in strategy formulation and implementation; CHD senior managers help shape strategy set by outside parties but leave it to NZD to implement • The NZD CEO keeps pushing or arguing with CHD managers until they get an agreement because they know that if they do not agree with him, he will go to see their CEO. In contrast, the CHD CEO believes the NZD CEO is young and ambitious but needs to grow up and thus needs support from him to get his job done • NZD managers believe CHD senior managers want MILK to be their cheap resource suppliers of infant milk powder exclusively for the Chinese market and grow at an unsustainable rate, whereas NZD wants to target a wider range of premium priced products to a number of international markets at a lower growth rate • CHD managers perceive NZD managers lack experience in China so that they alone should be responsible for designing strategies, which should centre on high volume and quality, but low-cost infant formula supply • NZD senior managers decide to maintain MILK as a low volume dairy factory for CHD to supply the Chinese market, but develop their own premium products for non-Sino international markets (e.g. UK, USA); CHD senior managers eventually accept MILK will supply lower volumes of dairy products, so focus on maintaining product quality and supply	• Different external constraints on corporate strategy independence • Different views on internal strategy process responsibility • Competing strategic goals	
• Other than irregular board meetings, the IJV partner companies never set up any specific inter-firm senior and/or middle management liaison meetings either in New Zealand or in China • The IJV partners both appear to avoid contact because: (1) Senior managers from both IJV partner companies do not talk to each other either before or after board meetings; and (2) Senior managers from both partners feel disappointed about their counterparts’ attitudes and performance, but do not make any real effort to solve emerging conflicts, preferring to keep their distance	• Lack of collaborative contact	Sensemaking about communication and trust
• In the absence of formal liaison mechanisms or interpersonal relationships between managers at any level, information is not passed on between the two companies (e.g. Middle managers of CHD are required to deal with senior managers of NZD, who instead delegate these tasks to lower-level managers; CHD managers say their requests to NZD to provide product information or other small favours are ignored) • There is a lack of understanding about the cultural and sensemaking context outside technical language translation: NZD senior managers/directors say they do not care about maintaining CHD senior managers’ ‘face’ while frustrated CHD senior managers/directors feel their wisdom is not appreciated and they are not being listened to • NZD senior managers try to read CHD senior managers’ body language to help interpret conversations at board meetings when CHD senior managers stop talking or refuse to talk	• Misaligned communication	
• NZD senior managers do not trust CHD senior managers, so avoid contact, withhold information and use legal contracts to control their behaviour; while CHD’s senior managers think NZD managers are too naïve when doing business in China, choosing to stand back and wait for them to make mistakes	• Lack of mutual trust	

## Findings

Our first major finding was that participants made sense of their own performance by continually judging their practices favourably against those of their partner managers. Managers from both firms regularly used terms such as “ongoing learning”, “growing experience”, “expected results” and “historical achievements” to characterise (i.e., make sense of) their own performance, while “lack of experience”, “below our expectations” and “disappointing” described their counterparts’ performance.

Secondly, unlike other interpretive studies on IJVs narrating collaborative success (e.g. Gertsen & Søderberg, 2011), our case showed profound *sensemaking discrepancy* emerging between the two partners’ managers. This manifested as reported conflict between partners, characterized by a pervading sense of what we termed *disjointed collaboration* in terms of strategy performance. We define this as a state of compromised engagement where operations continued, but actors found mutual understanding problematic as their sensemaking became fundamentally misaligned. The analysis below characterizes sensemaking discrepancy between partners in terms of the four themes and constituent dimensions identified in Table 2: sensemaking about identity, learning and experience, strategizing, and communication and trust.

### Sensemaking about identity

The first theme concerned a strong sense of organizational identity in each parent firm, contributing towards conflicting assumptions about the fundamental role and purpose of MILK, its partners, products and managers.

#### Pride in parent firms

Managers from each partner articulated a strong sense of being and belonging towards their own parent company, manifested as pride but characterized differently in each firm. Jack, NZD’s CEO, said, “We had pride in what we had achieved” in the relatively short (12) years of NZD’s establishment. NZD’s identity concerned “Being proud of who we are” (Thomas, a senior manager), through “Believing in the excellence of our product” (Jack, a board director), and “Being smarter than our competitors” (Peter, a senior manager).

CHD managers also felt proud to be part of their parent company, but because of its longevity and heritage (64 years) as a state-owned enterprise, in contrast to NZD’s more recent success: “Our rich history represented a precious cultural asset and we all felt proud to be a part of the company” (Li, CEO).

This strong sense of belonging was important in sustaining cohesion in each of the two parent identities, but inhibited MILK’s ability to develop its own distinct identity. As a suddenly-formed ‘child’ of two corporate parents, MILK had two organizational identities to reconcile rapidly. Unlike a single company subsidiary, it was unable to develop an identity organically over a longer time. Given more effective collaboration, a cohesive identity might have emerged at MILK, but dual identities persisted. These provided a foundation for sensemaking discrepancy, accentuated by other differences. Managers saw themselves as being on different ‘sides’, rather than part of a collaborative venture.

#### Misaligned expectations about MILK’s mission

Each parent’s cohesive sense of organizational identity conflicted with their view of MILK’s mission or organizational purpose. Success for NZD managers was characterized in commercial terms through a profit motive realised through differentiation as a provider of premium dairy products, innovation and expansion. Although NZD managers initially considered CHD an important shareholder bringing much-needed growth capital for MILK, the IJV was regarded more functionally as an investment vehicle. Cooperation with a Chinese parent was seen as unavoidable in penetrating the lucrative Chinese market and for later expansion in Asia. In NZD senior managers’ eyes, MILK was formed:

Thomas: “To bring us the money that we needed to grow our company”.

Peter: “To create more market opportunities by using our partner’s experience and networks”.

Although CHD senior managers saw MILK as an investment opportunity, this was more about acquiring quality, low-cost dairy products from an overseas partner to develop brands globally outside China than expanding domestically. MILK’s perceived mission was:

Gang: “To use efficient high-quality overseas resources for dairy products”.

Lu: “To develop global brands of infant milk powder with the IJV”.

CHD managers also emphasised a social mission for MILK, mirroring the positive working conditions CHD shared with other state-owned enterprises: A “Good work environment, good income and the good people here” (Lu). Senior CHD managers claimed their workforce enjoyed higher salaries than the industry average, and the ability to earn bonuses up to 10% or 15% of their annual salary. All staff members enjoyed free meals at the company cafeteria, transport to and from work, welfare benefits, and regular subsidized social gatherings. A strong sense of mission was encouraged by expectations about lifetime employment and an extended family culture. This contrasted with CHD’s intersubjective accounts of NZD’s far more commercial mission, as a middle manager explained:

Yan: “I thought of us like a family who should try to help each other, but they [NZD] don’t seem to think that way. They are much more focused on money rather than the relationship. Unless they need something from us, it’s like we don’t even exist in their eyes”.

#### Divergent cultural expectations

Partners’ contrasting identities were also partly attributed by NZD managers to differences in national cultural values. These were seen to filter into parent-company values and subsequently into MILK’s organizational culture through both sides’ managerial practices. NZD’s managers believed their parent company’s organizational culture was fluid, disorganized but fast-developing, partly reflecting the strongly individualistic and independent values they observed in NZ national culture. These in turn influenced their own managers’ values and behaviour at MILK. As a NZD middle manager’s intersubjective account revealed:

Colin: “[NZD] wants to run fast but hasn’t yet built its cultural foundation. It’s a huge risk, but I enjoy working for a company that isn’t so well organised and where there are lots of things still to be sorted properly”.

Researcher: “Why is that?”

Colin: “I like to be challenged, and I feel excited when working for a company where the systems, structures, and culture have not yet been fully developed because I like the feeling of being involved in building something”.

However, contrasting values were also linked to the pride/ mission identity characteristics above and perceptions of demographic differences; and these were articulated more strongly by both sets of interviewees than broad national cultural values. To CHD managers, MILK embodied a conflict between CHD’s family-orientation and NZD’s more aggressively-commercial orientation. CHD managers felt this caused NZD managers to disrespect them as part of a general lack of cultural appreciation. CHD managers believed their workplace benefits helped ensure employees worked long hours and were more committed to MILK, partly reflecting Chinese collectivist values, but also CHD’s work ethos; whereas NZD managers cared more about their personal lives, worked shorter hours and were less committed. This conflict became more apparent as the IJV developed, with the parents’ respective organizational cultures coming into sharp contrast: as a CHD senior manager related:

Feng: “Our culture is about people, harmony, and developing trusting relationships with our customers and partners, but they focus on money and achieving their own ambitions (Feng, senior manager)”.

Some NZD managers also quoted demographic differences between partner managers rather than differences in cultural values. NZD managers saw themselves as younger and more multi-cultural than those at CHD, observations confirmed by our demographic profiling. This showed the average age of NZD senior managers was in the early 40s, while middle managers were generally early 30s. CHD managers were on average 10 years older in both groups. Profiling also revealed that, although most CHD managers were New Zealanders, some were from Australia, Canada and the U.S. In addition, previous corporate experience may have influenced NZD culture, since some managers were recently recruited from another large NZ dairy company. Commitment to NZD’s organizational culture seemed more tenuous for these, with a higher rate of staff turnover evident. In contrast, CHD managers were more ethnically homogenous Chinese, albeit from different provinces, and had been at the firm most of their working lives.

Accounts revealed cultural differences intensified as NZD managers tried to extract greater profit from the sale of premium milk solids. NZD senior managers even claimed to be moulding MILK’s culture through their own corporate narrative by writing documents celebrating MILK’s achievements as a supplier of premium products. This was an intentional strategy practice to ‘Lift our company culture’ (senior manager Graham) and encourage sales staff to negotiate higher prices for NZD’s dairy inputs into MILK.

### Sensemaking about learning and experience

Interlinked with identity and cultural differences, partners’ learning and experience evidenced divergent sensemaking processes.

#### Contrasting approaches to work-based learning

NZD managers in the parent firm and MILK felt their individualism encouraged self-reliance and independent work practices. They believed this attracted employees able to cope with a stressful work environment and self-directed decision-making, but created uncertainty for those preferring a more supportive context. NZD senior managers received formal strategy training, but other managers accumulated lived experience by learning how to operate through on-the-job, self-training, often by making mistakes.

A NZD middle manager said colleagues “Preferred to work alone rather than work with others… [showing] unwillingness to help each other” (Joy). Accordingly, staff turnover was high, but managers became accustomed to this. As Karen, a HR manager, commented, “It’s a young company and we can get benefits by frequently getting fresh blood”. John, a middle manager, believed NZD valued experience obtained through on-the-job learning: “Working in a growing company enabled me to learn different things and continue to build my own experience”. Peter, a senior manager explained: “We made a lot of mistakes when trying to get the job done, but we learned a lot and improved our experience through day-by-day practice”.

In contrast, CHD managers valued learning from seniors in both parent and IJV. Inexperienced managers appreciated seniors’ hands-on knowledge, with two middle managers articulating, “Respect[ing] and trust[ing] our seniors” (Hui), “Valu[ing] teamwork and collaboration” (Yi), and a board director “Being cautious of making serious mistakes or repeating old mistakes. We also learned through practice, and thus the taught experience became our own experience” (Lu). Wei, a middle manager, explained: “Learning from our seniors was a quick way to increase our own experience and avoid repeating the mistakes that had been made in the past”.

Yi’s account evidences the role of risk-avoidance in learning-related sensemaking among CHD managers. This involved: (1) a very low tolerance for suffering heavy financial losses, either as a result of making new mistakes or repeating old ones; and (2) a relatively high tolerance for suffering small financial losses as a result of making new mistakes. Accordingly, less-senior managers learned from their superiors and were allowed to make small errors while being sheltered from more serious ones. This generally risk-averse learning was heightened by senior CHD managers having to account to Group and government superiors for their actions. Nested within multiple layers of accountability, CHD managers at all levels traded decision-making autonomy for security. Consequentially, many worked at CHD until they retired, with low turnover and expectations about exposure to risk. As Yi explained:

Yi: “When I’m responsible for a new assignment, I am not very concerned with making a new or small mistake involving the risk of suffering small financial losses… We have a rigorous procedure to finance a project, which involves the financial department checking the risks before any project is implemented. We also have monthly workshops where peers share their important learned experience”.

#### Misaligned management capabilities

NZD managers expressed a strong belief in their own capabilities, particularly marketing expertise, access to quality local milk resources and efficient processing technology. However, they regarded their Chinese counterparts’ management capabilities as inferior to their own, as the CEO stated:

Jack: “They neither have the ability to run the business nor the understanding of how business works in the international market, so we make all the decisions”.

The NZD CEO accused his Asian counterparts of a “naïve way of thinking” in terms of wanting to target the Chinese mass market rather than premium segments. MILK’s NZD directors criticised their boardroom counterparts, who attempted to issue direct orders to NZD executives to expand sales exponentially and expressed frustration when their lengthy justifications were rebuffed:

Peter: “They think they have the majority of the Chinese dairy market, and they naïvely think they can easily double or triple their annual sales, but they don’t have the ability”.

Jack: “The Chinese board directors like to lecture us at board meetings but we ignore their speeches and they then show their anger, which is very childish”.

NZD managers therefore ‘pushed’ CHD managers to accept their strategies, rather than engage in mutual knowledge sharing. Consequently, CHD managers said they found it difficult to intervene in strategic decisions. NZD managers also used supply contracts to specify the terms of engagement, perceiving their partners’ role mainly as *interference* with their own, ‘able’ decision-making. Accordingly, as senior manager Thomas explained, a central narrative became NZD’s senior managers *resisting interference* by CHD in their strategy-related practices:

Thomas: “The quality of their employees and their overall level of product technology is much lower than we expected, so I don’t think there is anything we can learn from them. Also, their way of doing business is different to ours… They always try to renegotiate to get lower prices even though they’ve already signed a contract… So, we just ignore their request and tell them what the deal is… They have no choice other than to take the deal at our price because we are their only supplier of infant formula”.

#### Lack of mutual learning due to competing senses of superiority

Each side believed collaboration was impeded by the other’s strong but unfounded sense of superiority. According to a CHD senior manager, NZD managers had “‘superior attitudes” and were too “egotistical” in ignoring CHD suggestions:

Lu: “They think they are better than us in all aspects, and so don’t like to listen to us even though we have shown our willingness to help them”.

In his intersubjective account, CEO Li reflected that this sense of superiority characterized NZD managers, despite some improvement in relations after NZD leaders travelled to their China-based facilities:

Li: “From the beginning of the collaboration, they just looked down on us, did what they wanted and often didn’t even listen to us. So, we took their senior executives on a tour of our company and showed them our advantages. After that, they started to listen a little more and the relationship seemed to get better”.

However, this competing sense of superiority continued, compromising middle managers on both sides when dealing with those higher up. For example, Hui (a CHD middle manager) believed Thomas (a senior NZD manager) failed to respond to her emails about a damaged shipment because Thomas did not want to communicate with a junior CHD manager. Kevin, a NZD middle manager, believed his own senior management was withholding operational information from CHD as a deliberate tactic. In fact, Kevin turned the ‘superiority’ argument around, maintaining it was CHD senior managers who had inflated self-perceptions, prompting them to “interfere” unnecessarily in operational issues:

Kevin: “The [CHD] managers seem to think their company is bigger and better than ours, and thus they believe that their management team is somehow superior to ours, but our senior managers don’t see it that way. This seems to have led to poor communication between the companies and complaints from both sides about the other’s poor contribution and performance”.

Overall, learning differences between the two sets of managers were exacerbated by contrasting beliefs in each other’s capabilities and a competing sense of superiority. These contributed substantively towards sensemaking discrepancy, with managers developing contradictory approaches. Senior NZD managers allowed their more autonomous subordinates to make decisions involving more risk. They found their partners’ hierarchical practices difficult to comprehend, seeing even operational decisions constantly referred upwards by middle and junior managers. In contrast, senior CHD managers expected to be involved in all decisions, partly to account to Group and government superiors for their decisions. However, NZD senior managers made little sense of CHD’s teamwork-based, internally-deferential learning approach.

### Sensemaking about strategizing

The third theme was sensemaking discrepancy arising more directly from strategizing approaches and priorities. Partners saw IJV equity holdings and contracts very differently and enjoyed contrasting degrees of strategic autonomy. They also had different views on who was responsible for which aspects of the strategy process, but both regarded strategy as a competitive arena, rather than a cooperative opportunity.

#### Contrasting perceptions of equity and contractual practices

Equity holdings, shareholder agreements and trading contracts were a major source of sensemaking challenges. In terms of equity, CEO Li claimed CHD always “Wanted to have a majority shareholding in the IJV”. At MILK’s formation, it was agreed to allocate equal equity stakes to the two partner firms, but after two months of subsequent negotiation, NZD agreed to sell a 51% shareholding in MILK to CHD, which appointed four IJV board members to NZD’s three. This temporarily resolved the ownership issue, but as the alliance coalesced, different perceptions about the centrality of shareholdings became linked to conflicts about identity, management capabilities and strategic control.

NZD managers believed a dilution of their shareholding would have little effect because their counterparts had “Poor understanding of Western ways of doing business” (Jack, CEO), particularly in relation to written contracts, which NZD saw as legally binding and defined both parties’ mutual obligations more than equity stakes. Chinese managers instead believed relationship-oriented business practices and verbal agreements coupled with a majority shareholding were more important than written contracts. NZD managers found it difficult to make sense of this relational view of contracts, believing collaborative problems arose from their partner’s lack of legal understanding: “For us, contracts are standard business practices on which trust is built, but for them it’s not the same” (Thomas). Once the IJV was established, the shareholders’ agreement constrained CHD’s selection of board members. In addition, ‘We used trading contracts to avoid renegotiations of price and/or other issues’ (Peter) and these “Decided how much infant formula we would produce” (Thomas). NZD managers commonly expressed the idea that their Chinese colleagues had much to learn about contractual practices, as Jack, a NZD and MILK board director, explained:

Jack: “The [CHD] people are very naïve. They don’t have a deep understanding of how documents work, but in our company, documents matter and are central to doing business”.

When further investment was needed in the IJV, a deteriorating relationship with NZD made CHD reluctant to contribute additional equity. Consequently, NZD senior managers renegotiated their stake to reduce CHD’s shareholding from 51 to 39 per cent, which the Chinese parent reluctantly agreed to. NZD managers felt this was a major strategic mistake by their counterparts because this would allow NZD greater board level control on top of contractual terms. Paradoxically, despite these new ownership arrangements, CHD senior actors increasingly felt MILK was *their* overseas subsidiary, rather than a strategic alliance. CEO Li believed “We were fooled into signing their contract, allowing them to manipulate the IJV”. He regarded NZD senior managers as having broken promises to significantly increase sales volumes and develop new products specifically for the Chinese market. When financial returns remained poor, CHD managers concluded their partner had inferior work standards and management skills. To cope with disappointed expectations, CHD managers came to regard MILK as a mere factory ran by NZD rather than a jointly-owned venture, since it had not developed an independent structure, management team and set of employees. Such conflicts helped grow and perpetuate the sensemaking discrepancy.

#### External constraints on corporate strategy independence

As indicated, the hierarchical nature of CHD’s internal strategizing involved Chinese directors deferring first to their corporate state-owned Group and ultimately to government officials. This encouraged the risk-averse learning behaviour examined earlier because of a complex layering of accountability. CHD managers substituted strategic decision-making autonomy for security, lifetime employment and social and welfare goals. Strong external accountability was ensured by CHD board members also being ministry officials. Well-aware of political implications, CHD managers were understandably reluctant to discuss their relationships with state officials during interviews. Instead, as a senior manager obliquely stated, they deferred to corporate Group policy when asked about constraints to their strategy independence:

Sun: “We kept our strategic orientations in line with that of the Group”.

In contrast, NZD senior managers were unconstrained by a corporate group structure or wider political forces, as the CEO stated:

Jack: “We focused on the vision of where we wanted to position ourselves…We owned and operated the company, so we made all the decisions”.

#### Different views on internal strategy process responsibility

Differences in strategic autonomy also contributed to sensemaking discrepancy about the roles and responsibilities of managers in MILK’s internal strategy process. Since their strategic priorities were largely shaped externally, senior CHD managers paid little attention to formal strategy practices involving strategy workshops or regular board meetings. The IJV board met infrequently (twice-yearly), more as a symbolic undertaking than a decision-making forum; and NZD managers felt their counterparts contributed little of substance at these events. Infrequent meetings were rationalised by CHD managers in terms of the inconvenience of long-distance travel and NZD managers initially being “Good honest people” (Lu, senior manager) who could be relied upon to implement strategy already imposed by outside forces. CHD’s leader explained a lack of direct involvement in formal MILK strategizing:

Li: “We focused on the strategies required to achieve the expected results rather than on the details of the step-by-step progress”.

A hands-off rationale was echoed by a CHD senior manager, who claimed:

Lu: “We took responsibility for strategic planning and guided them [NZD] to get the job done”.

CHD senior managers saw their strategizing practices at MILK as pragmatic, informal and action-oriented. As a senior CHD manager stated, during the intersubjective account phase:

Hui: “There is no strategy until there is something you can use to achieve the expected results. In other words, if a designed or planned strategy is not useful or helpful in achieving a target, it’s just an immature idea that is still waiting to become strategy. We focus on actions because we believe that actions produce pragmatic strategies rather than fanciful ideas”.

CHD senior managers delegated strategy implementation to their MILK middle managers, since goals were already given to them by external stakeholders:

Lu: “We implemented the strategic ideas formulated by our senior executives as well as developing them through practice”.

While senior CHD managers did not have specific strategy meetings about MILK, we observed weekly strategy meetings between NZD senior managers rationalized by CEO Jack as “Blueprinting our future ten years ahead”. NZD executives attended regular consultant-led workshops to formulate 10-year vision and positioning statements for the IJV. Jack observed, “Spending about fifteen hours per week at meetings caused us huge time pressure to get our work done”, but regarded this as important. Clearly, senior management had a much more formal, hands-on view of strategizing than their Chinese counterparts.

#### Competing strategic goals

Contrasting views on strategy content added to the sensemaking gap above concerning the strategy process. Formal agreement on MILK’s strategic goals did not happen during negotiations to establish MILK or afterwards. Consequentially, both parties pursued their own objectives, making it difficult to make sense of MILK’s overall direction. NZD executives aimed to develop Asian markets with premium-priced dairy products, focusing MILK on lower-volume production and higher quality. CHD leaders instead saw MILK as a factory for producing high-volume, low-priced dairy products for Chinese consumers. A senior NZD manager summarised this strategy contradiction:

Thomas: ***“***They want us to become their cheap resource suppliers, but we want to sell our products at a higher price, so infant milk powder is the only business we transact with them now” (Thomas, senior manager).

Senior NZD executive Peter articulated a basic conflict between low cost (price) versus differentiation (premium-pricing) strategies. CEO Jack also felt CHD leaders were naïve in an “unrealistic” desire to increase Chinese sales exponentially. Instead, NZD executives wished to take strategic advantage of consumer concerns with product quality and safety, as a result of a well-publicised infant formula poisoning at the market-leading Sino-NZ IJV^2^. NZD managers emphasised the need to grow MILK at a sustainable rate and provide tailored, “right fit”, innovative products for customers, while their CHD counterparts were more concerned with mass market growth. Peter, a NZD senior manager explained:

Peter: “The business is now five years old and we have enough experience to know where to target our business and which customers are the right ones for us. We are able to demand a higher price for our milk powder and we target those customers who are prepared to pay that higher price. If customers don’t fit this criterion, then we don’t want to do business with them”.

NZD’s CEO explained during the intersubjective account phase why he refused to communicate with CHD middle managers if they did not comply with his objectives:

Jack: “They only focus on the Chinese market and want us to become their overseas resources supplier, but it’s not what we want. We want to develop our company in the international market, so we’ve kept arguing about this issue since we established MILK… At the end we just ignore them and do what’s good for us. If they disagree with what we are doing or plan to do, we send it to the board for them to make a decision, and then we just push them until they agree with us”.

Eventually, NZD senior managers became disillusioned with what they saw as CHD personnel’s poor sales performance and MILK’s resulting low market share in premium milk solids. NZD executives decided MILK should continue supplying lower volumes of milk solids to their partner, while NZD pursued its differentiation goal in other international markets.

CHD managers also came to recognise these divergent objectives, with CEO Li accepting the IJV would not be able to become a mass-market producer. It began focusing on maintaining quality and delivery of a more limited product base instead of co-branded products. Li emphasized the need to *maintain harmony* in the IJV rather than have supply disrupted. However, his general feeling was of disappointed expectations arising from misaligned organizational purposes:

Li: “I am only concerned about product quality and whether or not they can deliver on time. Other things are none of my business. As long as there is no problem with the product quality, we won’t give up on them; otherwise, we can always choose to either sell our shares or use our authority in the IJV to change the CEO”.

Although beginning optimistically as a venture to which both parties showed commitment, MILK entered a Limbo-like state as it matured and its partners’ strategic objectives became increasingly irreconcilable. The venture continued to supply milk powder, but it was no longer functioning effectively as a collaboration.

### Sensemaking about communication and trust

The fourth theme concerned how lack of mutual contact at all levels, misaligned communication, and mistrust contributed to sensemaking discrepancy between partners.

#### Lack of collaborative contact

The relationship between MILK partners was characterized by a lack of formal and informal contact. Other than biannual board meetings, there were no working groups, committees or other liaison mechanisms to facilitate bilateral contact, as a NZD middle manager reflected during the intersubjective account phase:

Kevin: “Since I’ve worked here, they’ve never sat down together and tried to find a solution to smooth the trading process and get things working more efficiently”.

Neither were there strong personal working relationships between partners’ senior managers, with NZD executives admitting to preferring social distance from their counterparts. Directors did not engage in conversation before or after board meetings and, despite articulating disappointment at their partner’s perceived performance, neither party invested in individual or collective effort to build bridges. Neither admitted they could have performed better or that their own actions contributed to issues, instead blaming the other party for emerging problems. One CHD senior manager insisted he saw problems developing but rather than try to solve them chose to ‘Sit back and watch’(Lu) to maintain harmony. CEO Li eventually concluded:

Li: “Their [NZD and MILK’s] managerial and operational issues are none of our business”.

#### Misaligned communication

When they tried to communicate, both parties had trouble making sense of each other, including at board level:

Jack: “Their way of operating the business doesn’t fit with ours, so I don’t listen to them at board meetings and I know they [CHD’s board directors] are not happy”.

Language issues were certainly one reason for this: NZD managers complained about a lack of English competency among their opposites, directing Mandarin-speaking NZD middle managers to talk directly to CHD executives, despite the latter’s hierarchical status. Feeling ignored by their NZ counterparts and protecting their status, CHD senior and middle managers encouraged their junior staff to liaise with NZD managers. Ultimately, CHD’s CEO felt his managers’ lack of English fluency contributed towards NZD managers’ sense of superiority:

Li: “They look down on us and like to complain that our senior managers don’t speak English and are not committed to the development of the IJV”.

Even after spoken and written texts were translated, communication issues persisted. During inharmonious MILK board meetings, NZD directors saw their Chinese counterparts launch into long soliloquies and occasionally into what they believed were emotional outbursts. CEO Jack felt these were mainly for effect in signalling authority. CHD directors even banged their fists on the board table, explaining retrospectively that this was in frustration at not being listened to by their NZ colleagues. However, Jack felt it was easy to second-guess Chinese directors’ true meanings by observing their body language:

Jack: “The Chinese people have very rich body language and are not good at hiding their emotions, so I can easily read their body language and guess their meanings when they talk to each other in Chinese. I also ask my interpreter [an expatriate Chinese National fluent in both Mandarin and English] to quietly make notes for me about their group conversations”.

Most NZD middle managers felt messages were less obvious. Even ethnic Chinese-speaking NZD managers said they often experienced difficulty communicating with their CHD counterparts, especially in situations placing them between senior managers from both sides. As earlier examples showed, NZD senior managers viewed contract terms as fixed, whereas Chinese partners saw them as open to further negotiation. This meant delays and convoluted decision processes as terms were repeated, answers chased and information passed up, down and between each decision hierarchy. Jennifer, an ethnic Chinese NZD middle manager complained her CHD colleagues tried to change the terms of a milk powder shipment after it was agreed. As a result, she felt stuck in Limbo:

Jenifer: “I asked our warehouse to organise the shipment, but when it was ready and I asked them to complete the deposit, they suddenly wanted to change the packaging size and quantity…I talked to my manager who asked me to take responsibility for negotiating with them, but insisted that I shouldn’t change the contract terms… I felt frustrated because I was expected to stand in the middle… Because neither of them was willing to compromise, this order took about four months to complete”.

Meanwhile, CEO Li argued “Their [NZD] management was disordered and chaotic” and “their key leaders’ *qing shang* [interpersonal skills and capabilities] were poor” (Li). Senior manager Lu agreed:

Lu: “We focused more on people, while their focus was mainly on achieving their business ambitions”.

An ethnic Chinese NZD middle manager summarized the overall situation in terms of inadequate mutual understanding about communication norms, rather than just technical translation issues:

Joy: “I’m Chinese but I don’t understand them… Communication is not about whether or not we can understand or talk in each other’s language, but it’s actually about how we listen and how we talk”.

#### Lack of mutual trust

Finally, both parties indicated a lack of mutual trust. This was partly because of different views over written contracts, as Thomas’ extract earlier showed. Senior NZD manager Peter related this breakdown of trust to CHD attempting to renegotiate supply contracts even after they had been agreed:

Peter: “It was frustrating when they [CHD’s key actors] renegotiated with me some issues that have been discussed and included in the contract; so, I stopped them whenever they tried to renegotiate with me”.

Contracts were not the only source of mistrust. CHD senior managers felt they were not trusted by NZD colleagues to take part in MILK strategy decisions, with a director reflecting on CEO Jack during the intersubjective account phase:

Lu: “When I tried to talk to him about how we should develop our strategy, either at board meetings or when we had a private dinner together, he didn’t want to listen. I also found that if I tried to get close to him, he would suddenly become indifferent and keep his distance. I think he has a problem with regard to listening to and trusting people. I asked him if he still wanted us to be a shareholder and his answer was that he still wanted us to sit at the board but he didn’t want us to interfere with their work or decisions. Now we just keep the peace and stay back watching and waiting for him to make a big mistake, so we can teach him a lesson”.

Overall, partners found it difficult to make sense of each other’s shared concepts about identity, learning and experience, strategizing, communication and trust. The next section draws out the implications of the findings, to develop a theoretical framework grounded in study data (Eisenhardt & Graebner, 2007).

## Discussion and conclusion

The literature review identified a large gap in international management research on strategy-related sensemaking. Our case study addresses this gap, revealing fundamental differences in the way IJV managers made sense of their own and their partner’s practices in performing strategy roles. Our research questions asked:

How do managers from different partners in an international joint venture make sense of their own and each other’s performance in relation to strategy practices during collaboration?

And

What are the effects of severe discrepancy between partners’ sensemaking processes on collaboration in the international joint venture?

We found managers compared their own perceived strategy-related performance with that of their partner managers. A fundamental *sensemaking discrepancy* arose when sensemaking of each other’s actions became increasingly misaligned. This severely damaged collaboration efforts, since managers were increasingly unable to make sense of each other’s strategy-oriented practices.

Our case shows how sensemaking about strategy performance plays an important role within the wider flow of strategy praxis, seen by practice theorists as ‘all the various activities involved in the deliberate formulation and implementation of strategy’ (Whittington, 2006: 619). SP theory already shows performing the strategic conversation is important for managers (Rouleau & Balogun, 2011). It also indicates when sensemaking challenges are not resolved, strategy-related action is limited or non-compliance occurs (Stensaker & Falkenberg, 2007). This mismatch was severe at MILK, manifesting as sensemaking discrepancy about organizational identity, learning, strategizing, communication and trust.

### Sensemaking about identity

**Fig. 1:**
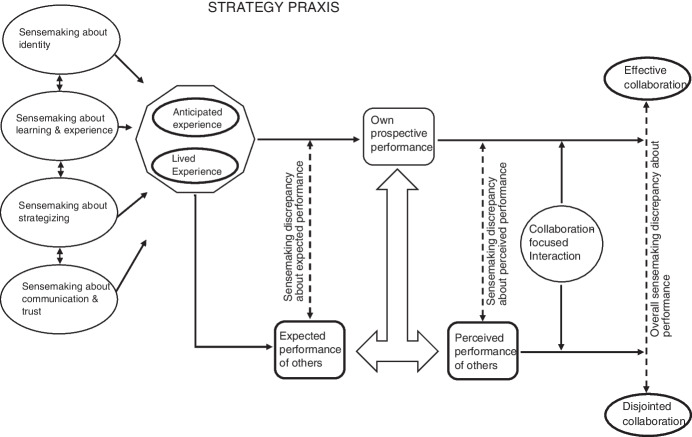
Sensemaking discrepancy about strategy-related performance

The first sensemaking category in Fig. 1 was strongly articulated by study participants. Identity is an integral aspect of sensemaking (Weick, 1995) and a basic issue here because it meant parent and MILK’s managers asking not only ‘Who are we?’, but also ‘Who are you?’ in relation to their partner managers. Although all organizations have multiple identities (Albert & Whetten, 1985; Pratt & Foreman, 2000), partners’ identities conflicted strongly, contributing to disparate sensemaking. While national cultural values are discussed in relation to IJVs (Barkema & Vermeulen, 1997; Pothukuchi et al., 2002), neither organizational identity nor national or individual-level identities are considered explicitly in IJV research. Our study shows this is a major research gap.

In fact, conflicting identities may be a particular hazard to IJVs, since they are composed of separate business entities trying to collaborate. Unlike wholly-owned ventures with a more cohesive identity, and acquisitions and mergers where one entity may subsume another (Moore, 2011), our study suggests parents’ identities may persist within an IJV as sources of potential conflict where collaboration is insufficient to promote greater cohesion. Each parent contributes tangible assets such as equity, technology, systems and personnel, but also intangible assets including a sense of organizational identity. As our case suggests, each identity is moulded by such influences as country of origin, location, stakeholders, national and organizational cultures, industry, organizational mission, history and experience. A sensemaking perspective on organizational identity offers a valuable contribution to IJV research, where asymmetries in tangible assets are insufficient explanations for MILK’s Limbo-like state.

A sensemaking view also extends analysis beyond cultural approaches. While culture theorists disagree as to the relative importance of the effects of national cultural values and organizational culture upon IJV practices (Pothukuchi et al., 2002), they do not directly consider the role of sensemaking upon organizational identity. MILK’s dual identities were juxtaposed rapidly and influenced by divergent managerial agendas and other influences. As a state-owned enterprise, CHD employees experienced a collectivist, social identity, in contrast to NZD’s private profit-focus, more dynamic working environment and greater individualism.

Ultimately, accounts showed both sets of managers identified much more strongly with their own parent firm managers - their in-group in social identification theory- than with their partners (their out-group) (Tajfel & Turner, 1985). They even acted as though they were competing *against* their collaborators. Actors from both sides shared a state of *nonidentification* with MILK: a cognitive disassociation state reminiscent of Limbo, where people seek neither active connection nor separation, or disidentification, from the organization (Kreiner & Ashforth, 2004). While NZD managers initially regarded MILK as a *joint* venture, both partners came to see it as their own subsidiary. Chinese managers noted MILK had not developed an independent structure, management or set of employees. However, data also suggested organizational pride, misaligned missions and divergent cultural expectations made managers’ sensemaking about identity more problematic.

### Sensemaking about learning and experience

Partners’ sensemaking about learning and experience also contrasted strongly. Again, such differences are not discussed in IJV research. Knowledge-based views see partners exchanging tacit and explicit (Grant & Baden-Fuller, 2004) and local knowledge (Makino & Delios, 1996), prior experience (Barkema et al., 1997) and sharing technological and financial risks (Park, 2011). Collaborators also establish rules and procedures to avoid one another’s opportunistic behaviour and protect their own knowledge (Liu et al., 2014), but we found sensemaking barriers to knowledge exchange also arise.

We saw NZD’s individualistic, on-the-job, error-tolerant learning as very different to CHD’s approach, characterised by managers’ deference to senior colleagues’ experience and low tolerance of mistakes. Each partner believed the other lacked management capabilities, while competing senses of superiority inhibited mutual learning. All were substantive contributions to a misalignment in learning-based sensemaking beyond existing knowledge-based explanations.

### Sensemaking about strategizing

Sensemaking discrepancy also arose from contrasting perceptions about equity holdings, contractual practices, external strategy constraints, internal strategy process responsibility and strategic goals. IJV scholars view equity structure as the most important design consideration (Choi & Beamish, 2004; Li et al., 2009), but this study suggests it is not only absolute equity holdings that are important but also sensemaking about these in relation to other strategic activities: each partner may believe they maintain strategic control, whether or not they hold a majority share.

Our findings contradict those arguing western partners often favour overall control of IJVs, while Chinese partners prefer to control technology transfer (Luo et al., 2001). Instead, NZD executives believed a majority shareholding made little difference because they controlled the IJV through contracts, in the absence of effective board meetings and liaison mechanisms. Perceptions of the importance of IJV ownership varied, depending on who is seen to control corporate activities (Madhok, 2006). While CHD senior managers aimed for majority ownership, their eventual acceptance of a minority holding showed they realised they had lost control of MILK. This indicates sensemaking about equity holdings and contractual practices influences IJV control in practice.

There were also clear differences in what strategizing meant to each partner. External institutional forces are important in directing Chinese state-owned-enterprises (Lin, 2020). Accordingly, CHD leaders took a hands-off approach in which they ‘guided’ strategy formulation, itself largely determined by Group and government policy; and avoided strong engagement with implementation issues. In contrast, NZD senior managers were involved in both formulation and implementation, through formal workshops and board meetings. NZD managers familiar with western-style, tools-driven strategy found it difficult to make sense of CHD managers’ informal, pragmatic idea of strategy – and vice versa.

Competing strategic objectives also created sensemaking discrepancy. Theorists stress the synergies enjoyed by parent firms combining resources and capabilities when sharing strategic goals (Beamish & Banks, 1987), and point to asynchronous behaviour when motives do not align (Hitt et al., 2000); but do not discuss the sensemaking implications of conflicting objectives. NZD managers believed CHD executives wanted MILK to be a fast-growing, high-volume dairy supplier for the Chinese mass market. Senior NZD managers instead saw MILK as a slower-growing, lower-volume premium producer for multiple Asian markets. Not only were mutual strategy objectives never formally agreed, but potential compromises were also not discussed. Consequently, partners struggled to make sense of MILK’s strategic direction.

### Sensemaking about communication and trust

Sensemaking discrepancy also arose through partners’ inadequate communication and lack of trust. This was not simply about technical language translation issues but arose from cognitive and behavioural differences (Hong et al., 2016). Language issues may create cognitive and emotional tensions in cross-national teams (Tenzer et al., 2014); but miscommunication resulted from failure to understand coded cultural information within instances of communication. For example, NZD’s senior managers were reluctant to ‘give face’ by responding respectfully boardroom ‘lectures’. CHD directors saw these as a means to impart wisdom about Chinese markets to their counterparts, but NZD directors dismissed these ‘long soliloquies’ as irrelevant, exacerbating boardroom tensions. CHD directors’ subsequent fist-banging showed frustration at their advice being ignored. In response, NZD directors claimed they could read CHD executives’ true intentions through their body language; whereas Chinese managers believed their New Zealand colleagues did not actively listen to them.

Research shows how asymmetries in language fluency can lead to an ‘us versus them dynamic’ in global teams (Hinds et al., 2014). Sometimes, multilingualism may be negotiated and a common corporate language emerge (Steyaert, Ostendorp, & Gaibrois, 2011). However, CHD managers felt NZD managers ‘looked down’ on them because of their lack of English language ability, compounding ‘us and them’ associations from strong parent company identities. This was exacerbated by a phenomenon reported in some emerging country alliances, where partners from contrasting central-planning and profit-oriented traditions experience *communication disengagement*, when facing a lack of equivalence between social and business concepts (Kuznetsov & Kuznetsova, 2014).

Building trust between partners is essential in effective governance, enhanced satisfaction and commitment (Beamish & Lupton, 2009) may be enhanced by a balanced equity structure (Madhok, 2006; Li et al., 2009), collaborative training initiatives and better communication (Kobernyuk et al., 2014); but none of these happened at MILK. Developing trust can be time-consuming and costly, since it cannot be created or destroyed instantaneously (Inkpen & Currall, 2004). It is also context specific, with an assertion of legal rights in China interpretable as a lack of trust in the partner (Child et al., 1997). This was evident at MILK, given partners’ contrasting views on the role of business contracts. Alongside conflicts over equity, mistrust developed over partners’ perceptions of each other’s capabilities. Overall, a lack of mutual trust between MILK’s partners heightened the sensemaking discrepancy.

### Own and others’ performance

The four sensemaking categories in Fig. 1 influenced managers’ perceptions of their own and others’ strategy-related performance. These impacted upon each manager’s *lived experience*: past, present and future self-taught learning about work experiences; and their *anticipated experience*: a vision or expectation in advance about what he/she might achieve at the IJV. A gap between anticipated and lived experience always exists, and this became a driving force helping the individual manager develop a sense of her/his *own (or self-) prospective performance*, or prospective sensemaking (Gioia, 2006; Ybema, 2010). In other words, in the course of the strategic practices undertaken in the IJV, people compared their own perceptions of how well they performed a task or role with these imagined, elevated outcomes.

Individuals then compared their own prospective performance with that of their partners in a dynamic process, also involving expectations and perceived performance. *Expected performance of others* refers to performance that actors anticipate of others who will actually perform a task or role. *Perceived performance of others* refers to interpretation of others’ **actual** performance in reciprocal interaction during the course of the IJV. These were compared with the individual’s own prospective experience, as shown by the triple-headed arrow in the middle of the diagram. In our case, a sensemaking discrepancy, or gap – the left dotted line- arose through comparisons between the individual’s own prospective performance and the sense made of others’ expected performance. A similar discrepancy emerged when comparing others’ perceived performance with what was expected and one’s own prospective performance – the subsequent dotted line. This led to two different, but related sensemaking discrepancies.

### Disjointed collaboration

In the early stages of collaboration, sensemaking discrepancy appeared to be more easily ignored by both partners. Over time, without action to address its causes, that discrepancy grew and became more intractable, leading to *disjointed collaboration*: a state of compromised engagement where the IJV continued operationally, but managerial collaboration became increasingly difficult as partners became increasingly unable to make sense of each other’s orientations and practices. In this Limbo-like state, the IJV continued to produce infant formula, but it was harder to repair or overcome the sensemaking discrepancy, institutionalising misunderstanding, miscommunication, and mistrust, as the perceived performance of partner managers failed to match expectations based on own prospective performance. Sensemaking in relation to identity, learning, strategizing, communication and trust continued to increase the discrepancy between own and others’ expected/ perceived performance.

### Effective collaboration and collaboration-focused interaction

We anticipate some sensemaking discrepancy is always likely to exist in an IJV, not only due to asymmetries over equity (Choi & Beamish, 2004), resources (Child & Yan, 1999), transaction costs (Beamish & Banks, 1987), interdependencies (Lioukas et al., 2016), knowledge transfer (Ho et al., 2019) and broad cultural values (Barkema & Vermeulen, 1997); but also in relation to sensemaking over identity, learning and experience, strategizing, communication and trust. In other reported collaborations (e.g. Jalonen, et al., 2018; Gertsen & Søderberg, 2011), sensemaking discrepancy also appeared, but was diminished through deliberate actions. In other words, *collaboration-focused interaction* – our conception of the sum of practices and inter-organizational relationships actors engaged in around collaboration - reduced the discrepancy between partners’ perceived strategy practices and led to more *effective collaboration*: the other state shown in the diagram. Although we have shown this at the end of the process, we anticipate such interaction may reduce discrepancy at any point. Ultimately, *overall sensemaking about performance*, the sum of the two discrepancies depicted, will increase or decrease along a continuum between two basic states of effective and disjointed collaboration. Thus, the more partners engage in collaboration-focused interaction, the less of a discrepancy there is likely to be between own prospective performance and others’ expected/ perceived performance. So, for example, had the two sides in the IJV sat down together, identified the root causes of the collaborative problems and tried to resolve them, there may have been a different outcome. Instead, partners blamed each other for the IJV’s poor performance and disjointed collaboration became more intractable. Ultimately, IJV managers became trapped in a Limbo-like cognitive state where they found it increasingly difficult to make sense of each other’s actions. The venture continued to manufacture dairy products, but operated ineffectively for both parties.

### Implications for theory

Our contribution to theory is twofold: 1) We identify four specific dimensions of sensemaking in our IJV based on identity, learning, strategizing, communication and trust; and (2) explain the process through which sensemaking discrepancy develops, based on novel concepts of sensemaking discrepancy about expected and perceived performance and disjointed collaboration. This help explicates Das and Kumar’s (2010a, 2010b) models of sensemaking *of chaos* and sensemaking *in chaos*, by providing a detailed practice-based cognitive mechanism to explain the processes by which those end states may occur. Sensemaking of chaos is akin to our state of effective collaboration, where predictability becomes more of an operating norm; whereas sensemaking in chaos to some extent reflects disjointed collaboration, where unpredictability is pervasive. However, our study suggests that, while sensemaking effects may be more pronounced in chaos, even in relatively predictable situations sensemaking is important. While successful acquisitions, mergers and alliances may resolve into a more cohesive identity (Moore, 2011), discrepancy may be more likely in IJVs, since they involve two or more partner identities juxtaposed in collaboration, often for an extended time. Cognitive conflict is likely, and perhaps even inevitable, between different schemas.

We extend theory about why conflicts may occur within IJVs beyond traditional explanations based on differences in equity, resources, technical knowledge and cultural values. Our finer-grained, sensemaking approach helps explain complex organizational phenomena. Although building on well-established cultural constructs - for example, respondents identified individualism in decision-making (NZD), and collectivism and deference to hierarchical authority (CHD) - we were able to examine how these apply in a particular context.

### Implications for practice

Given IJVs are at risk of developing sensemaking discrepancy, managers need to recognize the importance of sensemaking in influencing individual and organizational performance. The economic consequences (Tsang, 2016) of sensemaking discrepancy in IJVs are unknown. Prospective IJV parents need to account for sensemaking issues and consider whether scarce resources may be better placed in an alternative mode, such as co-marketing, research and development/ manufacturing contracts, a network relationship, or a wholly-owned subsidiary (Pan & Tse, 2000). In contexts like China, where government pressures to establish an IJV are more difficult to resist (Delios, 2017), a partner might consider moving towards a majority equity situation – or prepare to exit if sensemaking issues become too intractable.

If an IJV mode is selected, serious efforts must be made to understand and address sensemaking concerns before they intensify. Das & Kumar (2010a, 2010b) suggest in sensemaking of chaos situations, discrepancy may be manageable instrumentally through collecting more information, better conventional strategic analysis/planning and minor behavioural changes. However, our case suggests more fundamental interventions may be needed if discrepancy is not to become more intractable. Although in-depth access to a prospective partner’s sensemaking practices may be unrealistic during IJV negotiations, a more basic sensemaking ‘audit’ might be possible prior to signing contracts. This could examine potential sources of sensemaking discrepancy, and options for enhancing collaboration. These might include meaningful (i.e. not superficial) diversity training agendas for managers, focusing on sensemaking differences. Carefully structured, regular communication mechanisms may help sensemaking through corporate newsletters, videos, blogs and social media. Formal and informal liaison groups, workshops and taskforces involving all partners may usefully develop collaborative strategy. These and other initiatives may help generate shared understanding of sensemaking differences and resolve discrepancy before it intensifies.

### Limitations & areas of further research

Sensemaking accounts are, by definition, retrospective. Relying on post-hoc rationalisations is regarded by some as a perennial problem (Brown et al., 2008; Mills et al., 2010), but alternative methods invite other issues. Participants could keep diaries or journals of their IJV-related activities and perceptions, but these offer few real advantages over interviews because they may not capture their writers’ reflections any better. Document analysis is subject to the researchers’ values and interpretations, as in all ethnographic approaches (Moore, 2011). Observations are seldom in real-time as events unfold, and one cannot directly access actors’ sensemaking processing. This means we cannot totally escape actors’ own interpretations of their sensemaking processes, although intersubjective accounts help validate perspectives.

Of course, a single case represents only one context. Comparative cases and longitudinal analysis (Pettigrew, 1990) may increase understanding of the factors shaping sensemaking in relation to strategy practices. Future research could examine whether the four sensemaking dimensions here explain discrepancy patterns in other IJVs. Researchers may also develop testable propositions from our framework, and examine the mediating effects of variables upon sensemaking discrepancy and potential remedies, or collaboration-focused interaction. Studies could also explore how IJV participants interpret and interact with the external environment (Daft & Weick, 1984), or in contemporary forms of strategic collaboration, such as shared technology platforms, or vaccine development initiatives. One might also try to identify the salient cues considered when managers judge their partners’ practices.

Strategy scholars may be interested in whether tools and other artifacts influence sensemaking discrepancy. For example, SWOT analysis (Jarratt & Stiles, 2010) and PowerPoint decks (Kaplan, 2011) embed assumptions and routines about how strategizing ought to occur. It would be helpful to know whether tools reduce discrepancy by providing common, boundary-spanning frames to legitimize particular strategizing approaches. Other avenues include the role sensemaking plays in framing contests over strategy (Kaplan, 2008), and in producing strategy narratives (Brown et al., 2008), rhetoric (Erkama & Vaara, 2010), and broader truth effects (Ezzamel & Willmott, 2008).

Studies are also needed on the psychological and physiological effects of sensemaking discrepancy on IJV participants. We found evidence of stress when middle managers found themselves torn between the demands of senior managers from both sides in overseeing supply contracts. Psychological contract theory argues when mutual expectations over individuals’ perceived performance are not met, identity ambiguity, anxiety, distrust, organizational disidentification and ideological breach may occur, potentially threatening personal and organizational security (Thompson & Bunderson, 2003).

Our study has shown participants experiencing extreme disconnection with their partners. Research could establish whether there is a greater tendency towards disidentification (Kreiner & Ashforth, 2004) within IJVs than in other types of alliances. Scholars may examine whether such disconnection is with the whole organization or part of it and the form it takes. Researchers could explore how power around sensemaking is enacted in each parent organization; cultural variations in how identity work is undertaken; individual’s impression management when strategizing; and the degree to which leaders influence strategic change through sensemaking (Thomas et al., 1983). It is also important to know how strategic agendas in IJVs are established and materialized, and how ideological conflicts are resolved (Balogun et al., 2014).

Our study has generated a multi-dimensional framework explaining how sensemaking discrepancy may disrupt an international alliance. This led to a Limbo-like state part-way between collaborative success and failure. Milton’s (1667) ‘Limbo’ was a place for people and things forgotten. We use the metaphor to signify a venture seemingly lost in a state without strategic direction. Disjointed collaboration may be a staging post before organizational failure, or an ongoing state entrapping people and resources without a definite end. More work is needed to understand how IJV managers can make more sense of each other’s strategy-related activities.

## Data Availability

Raw data are not available because of confidentiality concerns. Assurances were provided to study participants and the organizations concerned that they would not be identifiable either as individuals or companies in any publications resulting from this study.

## Footnotes

1 All company and participant names are pseudonyms to preserve confidentiality.

2 In particular, the 2008 poisoning of 290,000 infants at the Sino-New Zealand Sanlu-Fonterra IJV. Chinese judges found Sanlu managers attempted to bulk-up infant formula with melamine, with the resulting scandal depressing consumer confidence in local brands and challenging regulators (Xiu & Klein, 2010).
